# The Imposing Front

**Published:** 1913-06

**Authors:** 


### The Imposing Front.

A few days ago we passed a building in process of construction and admired the massive stone walls of the lower story, suggesting security and stability for the whole superstructure. And then, as we looked upward, in spite of being reasonably well informed in modern methods, we experienced a shock of surprise to see a gap of a couple of stories, merely outlined in narrow lines of metal, and above it two or three stories of completed walls. Aside from the appearance, the solid stone walls might just as well have been glass windows and tin sheets. They had nothing to do with the strength of the building, which depended on the unobtrusive, seemingly frail beams of steel which would be hidden when the building was completed.

From having recently read, in the *Atlantic,* a praise for the old school reader, we were, perhaps, in a moralizing and philosophic mood, which we usually try to avoid. Anyway, we thought how well this building typified the medical profession—and others—with imposing, substantial men on the ground floor, putting up a good front to the passerby and apparently bearing the whole weight of the wisdom of the profession, whereas the real strength is due to the laboratory and other students, mostly concealed from public view, receiving little of the praise of the public for the stability of the whole structure. And in summer, with the hot air due to the reflection of the sun upon those massive stones on the ground floor adding oppressively to the impressiveness of the structure, the metaphor would be still more complete. Yet, in justice to those who usurp the gaze of the admiring throng, it is only fair to concede that, in times past, they really did carry weight and supported the professional pile, as did the literal stones.

